# Combination of Sensor Data and Health Monitoring for Early Detection of Subclinical Ketosis in Dairy Cows

**DOI:** 10.3390/s20051484

**Published:** 2020-03-08

**Authors:** Valentin Sturm, Dmitry Efrosinin, Manfred Öhlschuster, Erika Gusterer, Marc Drillich, Michael Iwersen

**Affiliations:** 1 Linz Center of Mechatronics GmbH, 4040 Linz, Austria; 2 Institute of Stochastics, Johannes Kepler University Linz, 4040 Linz, Austria; dmitry.efrosinin@jku.at; 3 SMARTBOW GmbH, 4675 Weibern, Austria; manfred.oehlschuster@zoetis.com; 4 Clinical Unit for Herd Health Management in Ruminants, University Clinic for Ruminants, Department for Farm Animals and Veterinary Public Health, University of Veterinary Medicine Vienna, 1210 Vienna, Austria; Erika.Gusterer@vetmeduni.ac.at (E.G.); Marc.Drillich@vetmeduni.ac.at (M.D.); Michael.Iwersen@vetmeduni.ac.at (M.I.)

**Keywords:** sensor fusion, ketosis, precision dairy farming, machine learning, time series classification

## Abstract

Subclinical ketosis is a metabolic disease in early lactation. It contributes to economic losses because of reduced milk yield and may promote the development of secondary diseases. Thus, an early detection seems desirable as it enables the farmer to initiate countermeasures. To support early detection, we examine different types of data recordings and use them to build a flexible algorithm that predicts the occurence of subclinical ketosis. This approach shows promising results and can be seen as a step toward automatic health monitoring in farm animals.

## 1. Introduction

Subclinical ketosis (SCK) is a common metabolic disease of dairy cows in early lactation, characterised by an increased concentration of ketone bodies in the absence of clinical signs of disease [1]. Analyzing the concentration of ß-hydroxybutyrate (BHB) in blood is the recommended reference test for detecting ketosis in dairy cows [2]. A commonly used threshold to define SCK is a ß-hydroxybutyrate (BHB) concentration in blood >1.2 mmol/L [3,4]. To detect SCK in dairy cows, various hand-held devices are commercially available, which were recently evaluated for use on farms [5,6]. The occurrence of SCK in dairy cows is associated with an increased risk of sequalae (e.g., clinical ketosis, displaced abomasum, metritis), decreased milk yield and impaired reproductive performance [3,7,8], affecting the economics of a dairy farm [9]. Major risk factors for the occurrence of ketosis are an excessive body condition score (BCS) before calving, an increased colostrum volume at first milking and an advanced parity [10]. Recent studies showed that subclinical and clinical diseases are associated with distinct animal behaviours, e.g., rumination as well as with standing and lying times, respectively [11,12]. Nowadays, more and more farmers rely on sophisticated sensor technologies for continuous and automated real-time monitoring of animal behaviours as well as of their health status [13,14]. The aim of this study was to predict the ketosis status of dairy cows within the first two weeks of lactation, based on 12 input variables, inter alia of the accelerometer system SMARTBOW (Smartbow GmbH, Weibern, Austria). The prediction is made using a flexible classification algorithm combining time series based acceleration data with other input specifically designed to cope with possibly different availability of data. In Section 2.1, Section 2.2 and Section 2.3, we discuss the different types of recorded data and how they were assessed. Moreover, we define our proposed algorithm and the main parts of it in Section 2.4 and Section 2.5. Section 3 contains the results from a statistical comparison of parts of the recorded data and the classification results. We conclude our work with a short summary and discussion in Section 4.

## 2. Materials and Methods

### 2.1. Animal Data and Sampling Procedures

Animal sampling and data collection were approved by the institutional ethics committee of the University of Veterinary Medicine Vienna, Austria (ETK-09/02/2016) as well as the Slovakian Regional Veterinary Food Administration. The study was conducted in 2016 and 2017 on a Slovakian dairy farm, housing approximately 2700 Holstein-Friesian cows. Animals were housed in ventilated freestall barns with group pens or cubicles, with rubber mats bedded with dried slurry separator material. To determine influences of barn climate and barn humidity on cows’ health status, climate loggers (Tinytag 2 Plus, Gemini Data Loggers Ltd., Chichester, West Sussex, UK) were installed in all groups. Temperature and humidity were automatically recorded and stored every hour. Animals were enrolled in the study at drying off, approximately 60 days prior to the expected calving date (D0) and followed up for at least 10 days, i.e., up to 10 days in milk (DIM). Blood samples were collected in week 8 (day −62 to day −56), 3 (day −21 to day −15), 2 (day −14 to day −8) and 1 (day −7 to day −1) before the expected calving date from a coccygeal vessel using vacuum tubes coated with a clot activator for serum collection (Vacuette, 9 mL, Greiner Bio-One GmbH, Kremsmünster, Austria). After clotting for a minimum of 30 min, samples were centrifuged [10 min, 18 ${}^{\circ }$C, 3000× *g*; (Eppendorf Centrifuge 5804, Eppendorf AG, Hamburg, Germany)] to harvest serum. Serum was stored at −20 ${}^{\circ }$C until further analysis at the Clinical Pathology Unit (CPU) of the University of Veterinary Medicine, Vienna, Austria. Samples collected in the week before parturition and at D0 were analyzed for non-esterified fatty acids (NEFA) at the CPU. At days 3, 5, and 8 of lactation, the BHB concentration was determined by use of an electronic hand-held device (FreeStyle Precision Xtra, Abbott GmbH and Co. KG, Wiesbaden, Germany), previously validated for dairy cows [6]. Animals showing BHB concentrations of >1.2 mmol/L were defined as suffering from subclinical ketosis and classified as ‘sick’. Body condition score (BCS) was visually estimated according to Edmonson et al. [15] and back fat thickness (BFT) was measured by an ultrasound device (Easy-Scan Linear, IMV Imaging, Meath, Ireland) as previously described by Schröder and Staufenbiel (2006) [16] in weeks 8 and 3 prior to calving and on D0.

### 2.2. Accelerometer

In this study, we used the accelerometer system SMARTBOW (Smartbow GmbH, Weibern, Austria), which was recently evaluated for monitoring of rumination [17,18] and detecting estrus events [19]. The system includes ear-tags (size and weight of 52 × 36 × 17 mm and 34 g) equipped with a three-dimensional accelerometer, receivers (SMARTBOW WallPoint) that are installed in the barn, and a local server (SMARTBOW Farmserver). Recorded data were sent from the ear-tags wirelessly and in real time to the receivers and transmitted to the local server, where data were processed by specific algorithms. In this study, 10 Hz sensors were used for measuring acceleration in three axes of head and ear movements of the animal with a range from −2 to +2 g. All cows were equipped with the sensor-based ear-tags approximately three weeks before the expected day of calving. In this study, the raw recordings were transformed into 7 data-streams that we are provided with. We inspect two time frames: the week before the day of calving and the the week after the day of calving. These seven data-streams represent the minutes per hour spent:

- lying/not lying,
- ruminating/not ruminating,
- inactive/active/highly active.

The time spent in either of the states in each category above add up to 60 min; thus, we converted the data into 5 dimensional data-sets for every individual, namely minutes per hour spent lying, ruminating, inactive, active, and highly active.

**Figure 1:**
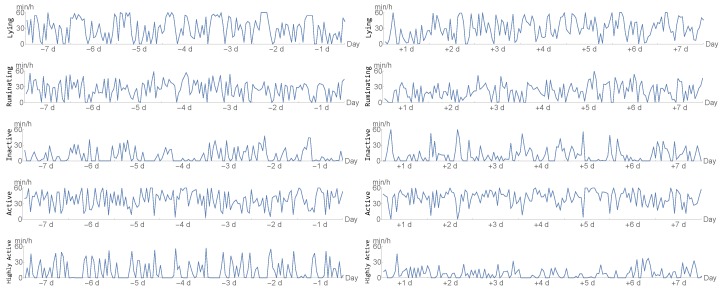
Depiction of the five time series for a single individual before (**left**) and after calving (**right**).

**Figure 2:**
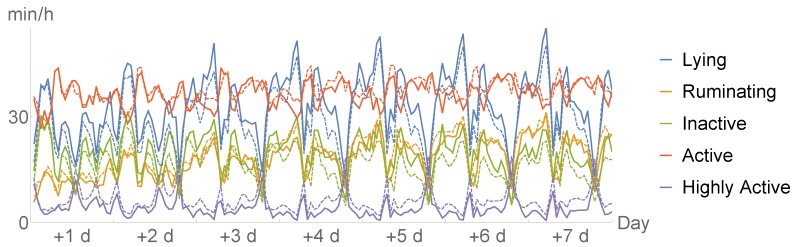
Time spent with respective behaviours after calving, averaged for healthy (dotted) and sick animals.

A example of such 5 data-streams for both the pre-partal and post-partal time frame can be seen in Figure 1 below. Moreover, we present the average time spent in the respective behaviours for sick and healthy cows after calving in Figure 2.

For thorough examination, a decision on how to tackle missing data, and some results based on the presented sensor data, we refer to [20]. A summary of the data can be found in Table 1.

### 2.3. Health Data

**Figure 3:**
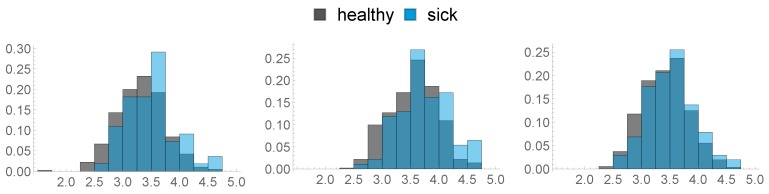
Histograms of the BCS at different times: 8 weeks prior (**left**), 3 weeks prior (**middle**), day of calving (**right**).

**Figure 4:**
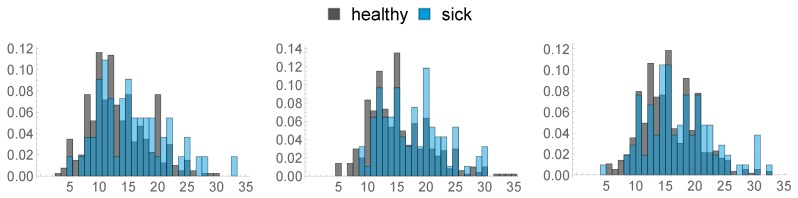
Histograms of BFT in mm at different times: 8 weeks prior (**left**), 3 weeks prior (**middle**), Day of Calving (**right**).

**Figure 5:**
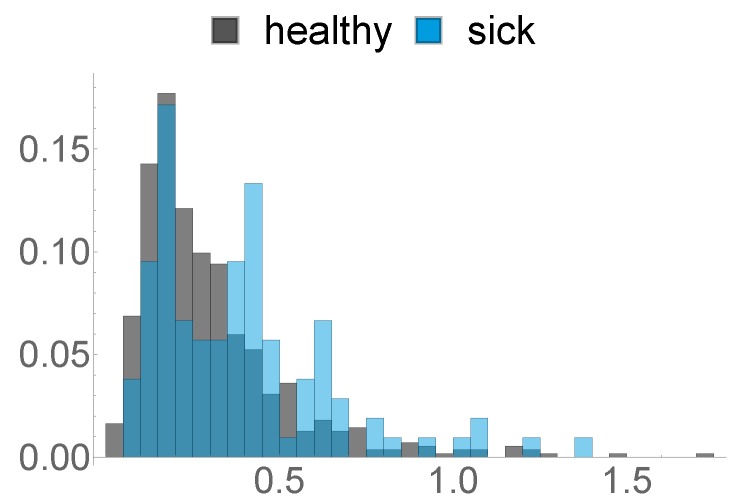
Histogram of the maximum NEFA value.

**Figure 6:**
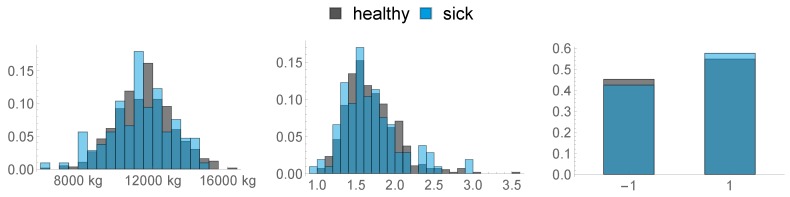
Histograms of features 8, 9, and 10.

**Figure 7:**
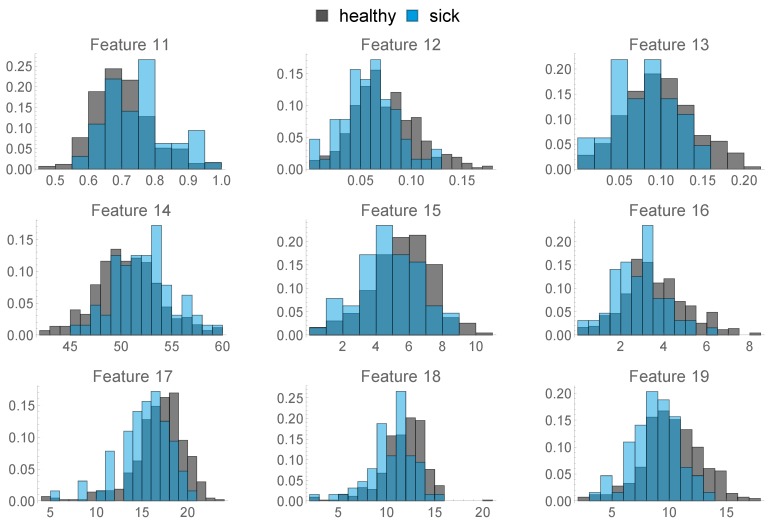
Histograms of features 11–19.

**Figure 8:**
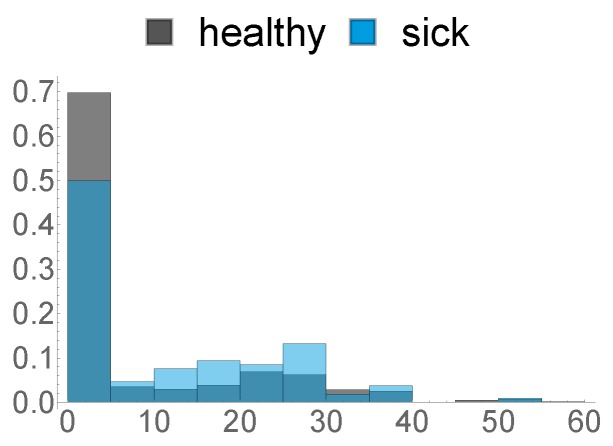
Histogram of the amount of time spent exposed to a THI of ≥72.

The second type of data we consider is health data either directly recorded on farm site or based on previous calvings. Measurements that are either ordinal or nominal are transformed into metric features. In the following list, the features that were assessed are described in detail:

- Body Condition Score (BCS): A total of three measurements were made, 8 weeks before calving (−8 w), 3 weeks before calving (−3 w) and on the day of calving (D0).
  1. −8 w
  2. −3 w
  3. D0
  In Figure 3, the distribution of these three features is visualised:
- Back Fat Thickness (BFT): As described above, three measurements were made:
  4. −8 w
  5. −3 w
  6. D0
  In Figure 4, the distribution of the BFT is visualised:
  7. Non-Esterified Fatty Acids (NEFA): We used the maximal NEFA Value of all measurements as described in Section 2.1. This feature is vizualised in Figure 5.
  8. 305 day Milk-Yield Equivalent: A measure that standardizes the milk yield of the previous lactation. Its impact on different diseases can be found in [21].
  9. This feature consists of the maximum observed fat/protein ratio during the previous lactation.
  10. Parity: We distinguished between primi- and multiparous cows and transformed these categories as follows: primiparous →−1, multiparous → 1.
    Feature 8, 9 and 10 are depicted in Figure 6.
- The following features are based on the locations the animals spent their time in the last two weeks before calving. We distinguished between three functional areas, namely cubicles (FA 1), feed alley (FA 2), and passageways (FA 3). These 9 features are depicted in Figure 7.
  11. Ratio of Hours spent only in FA 1
  12. Ratio of Hours where the animal spent more time in FA 3 than in FA 1
  13. Ratio of Hours where the animal spent more time in FA 2 than in FA 1
  14. Mean time spent per hour in FA 1
  15. Mean time spent per hour in in FA 2
  16. Mean time spent per hour in in FA 3
  17. Standard Deviation of Time spent per hour in FA 1
  18. Standard Deviation of Time spent per hour in FA 2
  19. Standard Deviation of Time spent per hour in FA 3
  20. This feature describes the amount of hours in the last week before calving, where the animal was exposed to a temperature–humidity index (THI) of 72 or higher, where a THI ≥ 72 is defined by the Austrian Chamber of Agriculture as “moderate heat-stress”, based on [22,23]. We can see the distribution of this feature in Figure 8 below.

The histograms above already give a first overview on to what extent the respective features differ between healthy and diseased individuals. A statistical comparison of all 20 features is discussed in Section 3.1.

### 2.4. Mathematical Section

This section covers the mathematical and algorithmic aspects for classifying the animals’ health status, i.e., we define the central elements that constitute our classification algorithm. As we are provided with two different types of input, namely time series and features, we utilise methods for both types.

Time Series Classification (TSC) is a non-trivial task, which is thematized in numerous publications. State of the art in TSC is ensemble methods based on transformations of the original series and using flexible distance measures. Simple approaches, such as using Nearest Neighbour algorithm with a suitable similarity measure, still yield comparative results [24,25]. Deep learning approaches are shown to be promising but are still outperformed by distance based methods [26]. As simple methods are desirable both for the simplicity and the comparative performance, we designed a flexible measure to quantify the similarity between two time series which builds the basis for the first step in classification:

**Definition 1** (*Distance Matrix* (DIMA))**.** *Letting* $a,b\in R^{n},f:R\times R\to R$ *and* $g:{\left\{1,\ldots ,n\right\}}^{2}\to R$ *with* n∈N *, we define the function* $D_{1}$ *:*

(1) $$D_{1}\left(a,b\right):=D_{1}\left(a,b;f,g,p\right)={\left(\sum_{i=1}^{n}\sum_{j=1}^{n}f\left(a_{i},b_{j}\right)g\left(i,j\right)\right)}^{\frac{1}{p}}.$$

Unfortunately, we can show that we have to oppose heavy limitations on the parameters to guarantee metric properties:

**Theorem** **1.** *Let* $a=\left(a_{1},\ldots ,a_{n}\right),b=\left(b_{1},\ldots ,b_{n}\right)\in R^{n}$ *and* $\delta_{ij}$ *be Kronecker’s delta*

$$\delta_{ij}:=\left\{\begin{array}{cc} 1 & if\;i=j\\ 0 & if\;i\neq j \end{array}\right..$$

*Consider the function* $D_{1}\left(a,b;f,g,p\right)$ *defined as in (1). With the choice*

$$f\left(a_{i},b_{j}\right)g\left(i,j\right):={\left|a_{i}-b_{j}\right|}^{p}\delta_{ij}=\left\{\begin{array}{cc} {\left|a-b\right|}^{p} & if\;i=j\\ 0 & if\;i\neq j \end{array}\right.,$$

$D_{1}$ *is a metric on* $R^{n}$ *for* p≥1 *and is equivalent to the n-dimensional Minkowski Distance [27]. More generally,*

$$g\left(i,j\right):=\delta_{ij}h_{i}$$

*with some weights* $h_{i}$ *that fulfil either:* $\forall h_{i}:h_{i}\in R^{+}$ *or* $\forall h_{i}:h_{i}\in R^{-}$ *are the only possible choices of g for* $D_{1}$ *being a metric on* $R^{n}$ *.*

**Proof of** **Theorem 1.** See Appendix A. □

Although the metric properties are of interest from a mathematical point of view and needed for some search speed-up algorithms [28], we can nevertheless utilise this function in a learning approach. As the general formulation of DIMA above allows for a variety of parameter settings, we assume that the function could be adjusted to build a central element for many other time series classification tasks. In our experiment, we decided on the following set of parameters:

$$p=1,f\left(a_{i},b_{j}\right)=\left|a_{i}-b_{j}\right|$$

The function g(i,j) is constructed as follows: given a training set of uni-variate data with two healthy and sick classes, with $n_{h}$ and $n_{s}$ elements in the respective classes:

$$X_{h}=\left\{x_{h,1},x_{h,2},\ldots ,x_{h,n_{h}}\right\}\;\mathrm{and}\;X_{s}=\left\{x_{s,1},x_{s,2},\ldots ,x_{s,n_{s}}\right\}$$

with

$$x_{h,i}:=\left(x_{h,i,1},\ldots x_{h,i,n}\right),\;i=1,\ldots ,n_{h}\;\mathrm{and}\;x_{s,i}:=\left(x_{s,i,1},\ldots x_{s,i,n}\right),\;i=1,\ldots ,n_{s}$$

we calculate the respective class means

$$x_{healthy}=\frac{1}{n_{h}}\left(\sum_{j=1}^{n_{h}}x_{h,j,1},\ldots ,\sum_{j=1}^{n_{h}}x_{h,j,n}\right)\;\mathrm{and}\;x_{sick}=\frac{1}{n_{s}}\left(\sum_{j=1}^{n_{s}}x_{s,j,1},\ldots ,\sum_{j=1}^{n_{s}}x_{s,j,n}\right)$$

and define the matrix *G*, where $I_{n\times n}$ denotes the n×n Identity Matrix:

$$G_{ij}=sign(\min\{|x_{healthy,i}-x_{sick,j}\left|,\right|x_{sick,i}-x_{healthy,j}\left|\right\}-\max\{|x_{healthy,i}-x_{healthy,j}\left|,\right|x_{sick,i}-x_{sick,j}\left|\}\right)$$

$$G\left(\lambda \right):=\lambda G+\left(1-\lambda \right)I_{n\times n}$$

$$\lambda \in \left\{2^{-i}|i\in \left\{0,\ldots ,15\right\}\right\}\cup \left\{1-2^{-i}|i\in \left\{0,\ldots ,15\right\}\right\},g\left(i,j\right):=G{\left(\lambda \right)}_{ij}$$

**Figure 9:**
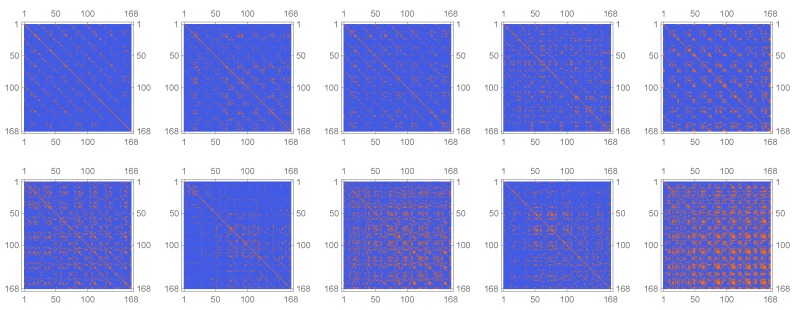
Depiction of the matrix G(0). Each column represents one of the five five behaviours. First, row: before calving, second row: after calving. Colouring: 1 = Orange, −1 = Blue.

Please note that, in case of λ=1, $D_{1}$ reduces to the Manhattan Distance with our choices. In Figure 9 below, we see an exemplary visualisation of the matrix G(0)=G for our two considered time frames.

### 2.5. Machine Learning in Animal Disease Detection

Machine Learning approaches have been heavily used in animal behavioural/disease assessment over the last few years. Take as an example [29], where the authors applied different methods for detection of subacute ruminal acidosis in dairy cows. In their results, k-Nearest Neighbors showed the best results, outperforming deep learning methods and decision trees. In [30], the authors test deep learning architectures for early detection of respiratory disease in pigs and compare them with classical time series regression approaches. Their results do not show any significant differences in performance measures of the presented methods. The authors of [31] used a wearable device and a one-class support vector machine algorithm to detect lameness in dairy cows.

Having introduced the first main element of our approach in the last sub-section, we continue with shortly describing another two central elements: We utilise Nearest Centroid Classification [32] using Function $D_{1}$ with G(λ) as above for TSC, and a Naive Bayes Classifier [33] for the feature-based classification. We decided on the NCC algorithm as it is simple and inherently avoids bias based on class frequencies. Moreover, we utilised the well-known naive Bayes algorithm, as it can be easily adjusted to handle missing features both in the learning step and while testing, as we can learn parameters on reduced examples, and just omit the probabilities for testing where a data-point is missing. Thus, our algorithm can be employed for 1 to up to 20 features available. In case of all features missing, one could introduce indecisive results, to indicate that the next steps are up to farm management, or classify solely based on ear-tag data. We omit the description of these two algorithms, and information can be found in the respective citations. Moreover, we decided on a feature-selection step using Relief [34].

We describe the tailor-made algorithm in the following section.

### 2.6. Proposed Algorithm

The first step is to split up the data into stratified sets for 10-fold cross validation. About 90% constitute the training data, on which the parameters are learned, while the resulting algorithm is used to classify the remaining ∼10 percent. The results are added up. Thus, we repeat the following steps 10 times:

1. For every data stream of the sensor data, we learn an optimal parameter λ such that the leave-one-out inner cross validation balanced error is minimised using an NCC with distance function $D_{1}$ with G(λ). In case of ties, the highest value of λ is chosen.
2. Using these 5 (possibly different) parameters, we assume an animal to be sick or healthy, if the five trained NCCs from Step 1 decided at least 4 out of 5 times for a certain class label.
3. The remaining examples are classified as follows:
  (a) The features are sorted according to the results from using Relief on the whole training set. For using this algorithm, we need a complete data-set where we only include examples in which all features are available.
  (b) Afterwards, we employ an inner 10-fold cross validation to find the optimal amount of features to take, starting with the ones ranked highest and consecutively add the following according to our ordering. The optimum is calculated with respect to balanced accuracy. In this step, we also only include training examples that are complete.
  (c) The features are finally processed using a Naive Bayes algorithm to classify the yet undecided examples.

The estimated labels are compared with the actual ones and the outcomes are added up to the results presented in Section 3.2.

## 3. Results

### 3.1. Statistical Comparison

In this section, we compare the features we defined Section 2.3 using significance tests. We employ a Two Sided Mann–Whitney U Test with a *p*-value of 0.05. We decided for a non-parametric test, as our data-set violates assumptions such as normality. Using Bonferroni Correction for multiple hypothesis tests, we arrive at a threshold for significant results of 0.05/20=0.0025. The Mean ± Standard Deviation (μ±σ) of each feature for both sick and healthy animals can be found in Table 2 below. Moreover, we added the exact *p*-values of each individual comparison, where a ${}^{*}$ indicates a significant difference.

Table 2 above shows that seven of our features do not differ statistically significant between healthy and sick animals, while 13 or 65% do. We can observe that the BCS before calving is higher for diseased animals, with a significant difference three weeks before calving, a finding that supports the results in [10]. In addition, in accordance with [10], the prevalence of SCK in multiparous animals is slightly higher, although there is no significant difference. Our results corroborate the findings in [35] where the authors also found a significant difference in NEFA concentration between healthy and ketotic cows.

All features based on location show significant differences, a result which we interpret as indicating that animals with higher risk of SCK tend to move less, i.e., variate their location less frequently than healthy cows.

In [36], the authors concluded that heat stress increases the ketosis risk mid-lactation. Our results point to the hypothesis that even prepartal heat stress may have an influence on development of SCK, as the average time spent under heat stress is significantly higher in diseased cows than in healthy ones. On the contrary, the authors of [37] calculated a 1.6 times higher risk of clinical ketosis in early lactation if the THI was lower than 83 on the day of calving in comparison to hotter days.

As we discussed the topic of possibly missing data, we briefly state the percentages of missing features below in Table 3.

### 3.2. Classification Results

As we assumed the location features to be rather specific, we evaluated our algorithm a total of four times, where we included the data described as follows:

1. Sensor data before calving, location features included
2. Sensor data before calving, location features not included
3. Sensor data after calving, location features included
4. Sensor data after calving, location features not included

Applying our algorithm using a tenfold cross validation yielded the following results: We start out with stating the confusion matrices, for the prepartal sensor data with location features included (left) and without a location (right):


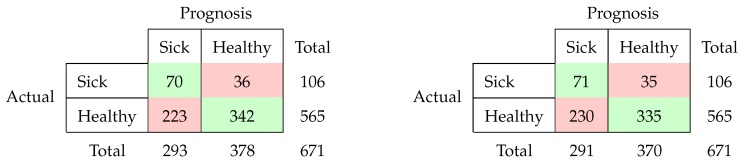


Moreover, we state the results when considering the acceleration data after calving below. On the left-hand side, we find the results when including the location data; on the right-hand side, the results when excluding them:


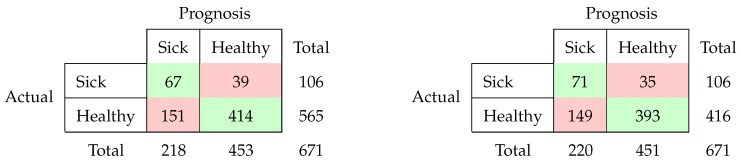


Based on these confusion matrices, we can calculate some famous performance measures, which we present in Table 4 below. The best values in each column are boldfaced.

The results, which clearly show better results for the post-partal time frame, are consistent with the findings in [20]. Although using pre-partal data seems desirable with respect to early detecting, the results indicate that the difference is more distinct post partum as all considered performance measures are the highest for Experiment 4.

Moreover, when comparing the difference w.r.t to the inclusion of location data, the results are slightly better when excluding the location data for both times frames, which is surprising given the statistically significant differences between healthy and sick cows as described in Section 3.1.

The percentage of correctly identified sick animals (=Sensitivity) varies from 63.2%–67.0% while the ratio of correctly identified healthy animals (=Specificity) varies from 59.3%–73.6%. The Negative Predictive Value is very high for all experiments (0.91%–0.92) but should be treated with caution as it is highly affected by an imbalanced data structure. As described in Table 1, we are dealing with an imbalanced class structure.

When looking at the results from Experiment 4, we see that more than two-thirds of the sick animals were detected correctly, while nearly 75% of the healthy animals were labelled correctly. Due to the imbalanced prevalence of class labels, estimating “sick” for a data set is only correct for about 32%, as can be seen by inspecting the precision, which still leaves room for improvement.

The algorithm proposed in Section 2.4 can be easily adjusted to include more individuals being classified as sick, useful e.g., when assuming the algorithm as a first “selection”. For that, we can learn a threshold in Naive Bayes such that a certain percentage of “sick” training examples is classified correctly. Moreover, we can only filter data-sets in the first step where all five NCC were decided for the same class label, which leaves possibly more examples without a definitive decision.

### 3.3. Parameters and Relevant Features Learned

As we estimated the classification quality in a cross validation scheme, we repeatedly selected possibly different subset of features. We state the results as we assume these choices reflect the relevance of the respective features. As we distinguish between two experiments each, where we either included or excluded location features, we have a total of 20 feature selection procedures for both scenarios.

**Figure 10:**
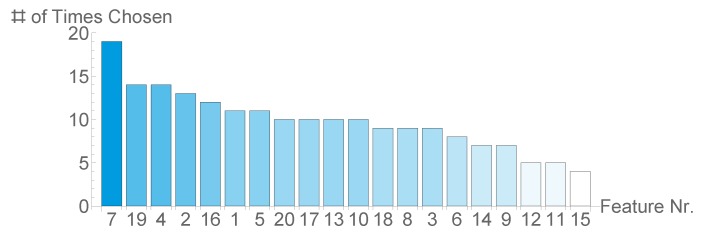
Bar-Chart with amount every feature was chosen in our inner cross validation step.

Figure 10 shows that the amount of times a feature was chosen varies greatly, as it ranges from 3 up to 19. NEFA was chosen in 19 out of 20 splits, an indicator of its relevance for detecting SCK. Moreover, all BCS and BFT values were chosen in more than a third of all splits. The three least chosen features are all based on location.

**Figure 11:**
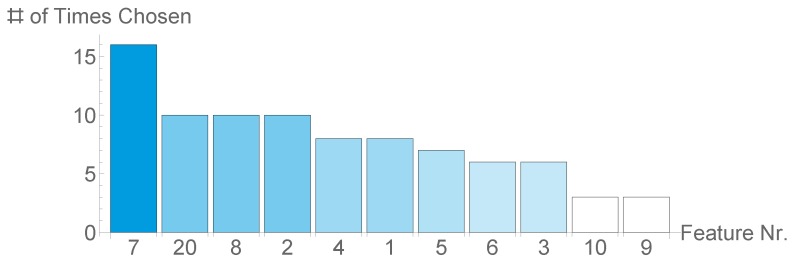
Bar-Chart with amount every feature was chosen in our inner cross validation step, when excluding the location features.

Figure 11 shows that, also when excluding the location features, the amount of times a feature was chosen varies greatly from 3 up to 16. NEFA was chosen in 16 out of 20 splits, followed by THI, Milkyield and BCS -3 w. Based on this observation, the Parity and Max f/p ratio can be considered as having low relevance.

## 4. Discussion

In this article, we presented results from a study to identify indicators for subclinical ketosis in dairy cows around calving. Moreover we constructed an algorithm, which aims for estimating the health status.

We included a statistical comparison of different parameters, based on milk yield and components, animal movements within the barn, ambient temperature, and on visual observation. The results showed significant differences in 13 of the examined parameters between healthy cows and ones suffering from SCK. A literature review showed that our results partly corroborate the conclusions from other studies.

In a second step, we introduced a flexible machine learning approach, which combines elements of TSC with classical feature based algorithms. It is designed to be simple, interpretable, and flexible with respect to data availability. The results show that our approach is a promising first step for automatic recognition of diseases in dairy cows.

Future work will include more elaborate machine learning approaches to tackle the problem of early detection of SCK.

## Figures and Tables

**Table 1:** Amount of healthy and diseased animals.

Health Status	Examples	Frequency
Healthy	565	84.20%
Sick	106	15.80%

**Table 2:** Statistical comparison of features. We state that the respective class means plus one standard deviation and the *p*-value when comparing using a Mann–Whitney test.

	**BCS -8 w**	**BCS -3 w**	**BCS Day0**	**BFT -8 w**	**BFT -3 w**
μ±σ healthy	3.19 ± 0.432	3.426 ± 0.428	3.298 ± 0.41	13.425 ± 5.002	15.393 ± 5.261
μ±σ sick	3.377 ± 0.446	3.613 ± 0.445	3.395 ± 0.424	15.527 ± 5.83	17.581 ± 5.261
*p*-value	0.00753024	0.000629145 *	0.0493177	0.0148332	0.000184263 *
	**BFT Day0**	**NEFA**	**305-D Milk**	**Max f/p Ratio**	**Parity**
μ±σ healthy	15.248 ± 4.846	0.296 ± 0.222	11538.3 ± 1528.18	1.686 ± 0.327	0.096 ± 0.996
μ±σ sick	16.892 ± 5.577	0.384 ± 0.256	11317.7 ± 1713.15	1.676 ± 0.372	0.151 ± 0.993
*p*-value	0.00630105	0.00015792 *	0.33808	0.449638	0.600132
	**Location1**	**Location2**	**Location3**	**Time Area 1**	**Time Area 2**
μ±σ healthy	0.703 ± 0.09	0.074 ± 0.032	0.098 ± 0.042	50.658 ± 3.188	5.564 ± 1.853
μ±σ sick	0.748 ± 0.093	0.059 ± 0.026	0.08 ± 0.037	52.285 ± 2.96	4.705 ± 1.857
*p*-value	0.00020492 *	0.000434685 *	0.00242925 *	0.0000765469 *	0.000498528 *
	**Time Area 3**	**SD Area 1**	**SD Area 2**	**SD Area 3**	**Hours THI Greater 72**
μ±σ healthy	3.563 ± 1.478	16.821 ± 2.937	11.711 ± 2.309	10.233 ± 2.577	6.981 ± 11.911
μ±σ sick	2.825 ± 1.155	15.336 ± 2.811	10.538 ± 2.374	8.799 ± 2.039	10.858 ± 12.576
*p*-value	0.000151992 *	0.0000174387 *	0.0000541604 *	0.0000066027 *	0.0000583004 *

**Table 3:** Percentages of features missing.

Feature	1	2	3	4	5	6	7	8	9	10	11–19	20
% Missing	31.45	11.03	1.19	31.45	11.03	1.19	1.79	0.15	0.00	0.15	26.23	0.00

**Table 4:** Performance Measures for our proposed algorithm for both time frames. Acc = Accuracy, Sens = Sensitivity, Spec = Specificity, Prec = Precision, J = Youdens Index, κ = Cohens κ, MCC = Matthews Correlation Coefficient, NPV = Negative Predictive Value.

Experiment	Acc	Sens	Spec	J	κ	F-Score	Prec	MCC	NPV	Lift
1	0.6051	**0.6698**	0.5929	0.2627	0.1504	0.3489	0.2359	0.1927	0.9054	1.6454
2	0.6140	0.6604	0.6053	0.2657	0.1548	0.3509	0.2389	0.1954	0.9048	1.6732
3	0.7168	0.6321	0.7327	0.3648	0.2553	0.4136	0.3073	0.2841	0.9139	2.3651
4	**0.7258**	**0.6698**	**0.7363**	**0.4061**	**0.2826**	**0.4356**	**0.3227**	**0.3155**	**0.9224**	**2.5399**

## Appendix A

**Proof.** We want to prove the following statements:

(A1) $$\begin{array}{cc} f\left(a,b\right)g\left(i,j\right)={\left|a-b\right|}^{p}\delta_{ij}\;\Rightarrow & \;D_{1}\;\mathrm{is}\;\mathrm{equivalent}\;\mathrm{to}\;\mathrm{the}\;n-\mathrm{dimensional}\;\mathrm{Minkowski}\;\mathrm{distance} \end{array}$$

(A2) $$\begin{array}{cc} D_{1}\;\mathrm{is}\;a\;\mathrm{metric}\;\mathrm{on}\;R^{n}\;\Rightarrow & \left(g\left(i,j\right)=\delta_{ij}h_{i},\;\mathrm{with}\;h_{i}\in R^{+},i\in \left\{1,\ldots ,n\right\}\right)\\ \; & \vee \left(g\left(i,j\right)=\delta_{ij}h_{i},\;\mathrm{with}\;h_{i}\in R^{-},i\in \left\{1,\ldots ,n\right\}\right) \end{array}$$

Let us begin with (A1): We plug into the definition of $D_{1}$ with our special choice for *f* and *g*:

(A3) $$\begin{array}{cc} D_{1}\left(a,b;f,g,p\right) & ={\left(\sum_{i=1}^{n}\sum_{j=1}^{n}{\left|a_{i}-b_{j}\right|}^{p}\delta_{ij}\right)}^{\frac{1}{p}}={\left(\underset{=0}{\underset{︸}{\sum_{i=1}^{n}\sum_{j=1,j\neq i}^{n}{\left|a_{i}-b_{j}\right|}^{p}\delta_{ij}}}+\sum_{i=1}^{n}\underset{=1}{\underset{︸}{\delta_{ii}}}{\left|a_{i}-b_{i}\right|}^{p}\right)}^{\frac{1}{p}}\\ \; & ={\left(\sum_{i=1}^{n}{\left|a_{i}-b_{i}\right|}^{p}\right)}^{\frac{1}{p}}. \end{array}$$

Since the last expression (A3) is equivalent to the *n*-dimensional Minkowski distance, we finished the first proof. Let us continue with the second statement (A2): First, we observe that (A2) is equivalent to the following implication:

(A4) $$\begin{array}{cc} D_{1}\;\mathrm{is}\;a\;\mathrm{metric}\;\mathrm{on}\;R^{n}\; & \Rightarrow \;g\left(\hat{n},\hat{m}\right)=0\;\forall \hat{n},\hat{m}\in \left\{1,\ldots ,n\right\}\;\mathrm{with}\;\hat{n}\neq \hat{m}. \end{array}$$

(A5) $$\begin{array}{cc} \; & \wedge (g\left(\hat{n},\hat{n}\right)>0\;\forall \hat{n}\in \left\{1,\ldots ,n\right\}\vee g\left(\hat{n},\hat{n}\right)<0\;\forall \hat{n}\in \left\{1,\ldots ,n\right\}) \end{array}$$

The proof of (A4) is trivial for n=1, as every statement with an universal quantifier (∀) on an empty set is true. The proof of (A5) is also trivial for n=1, since g(1,1) cant be zero, as this would imply M(a,b)=0 for all a,b. Therefore, let us continue proving (A4) by assuming n≥2: Suppose now $D_{1}$ is a metric on *n*, which means that the metric conditions have to hold. First, we derive some properties of *f*: We start with the observation that f(x,x) has to be 0 for all x∈R:

(A6) $$\begin{array}{c} f(x,x)=0\;\forall x\in R. \end{array}$$

Let $a={\left(x,\ldots ,x\right)}^{T}\in R^{n},x\in R$: From the *Identity of Indiscernibles*-property of a metric, we see:

$$\begin{array}{c} D_{1}\left(a,a\right)=0⟺D_{1}{\left(a,a\right)}^{p}=0⟺\sum_{i=1}^{n}\sum_{j=1}^{n}f\left(a_{i},a_{j}\right)g\left(i,j\right)=0⟺\sum_{i=1}^{n}\sum_{j=1}^{n}f\left(x,x\right)g\left(i,j\right)=0\\ \;⟺f\left(x,x\right)\sum_{i=1}^{n}\sum_{j=1}^{n}g\left(i,j\right)=0⟺\left(f(x,x)=0\right)⋁\left(\sum_{i=1}^{n}\sum_{j=1}^{n}g\left(i,j\right)=0\right). \end{array}$$

Next, we can easily show that

(A7) $$\begin{array}{c} \sum_{i=1}^{n}\sum_{j=1}^{n}g\left(i,j\right)\neq 0 \end{array}$$

has to hold under the assumption that $D_{1}$ is a metric. Therefore, let

$$a={\left(x,\ldots ,x\right)}^{T},b={\left(y,\ldots ,y\right)}^{T}\in R^{n},x,y\in R,x\neq y.$$

We know that $D_{1}{\left(a,b\right)}^{p}>0$ has to hold. Assume now (A7) as false:

$$\begin{array}{cc} D_{1}{\left(a,b\right)}^{p} & =\sum_{i=1}^{n}\sum_{j=1}^{n}f\left(a_{i},b_{j}\right)g\left(i,j\right)=\sum_{i=1}^{n}\sum_{j=1}^{n}f\left(x,y\right)g\left(i,j\right)=f\left(x,y\right)\sum_{i=1}^{n}\sum_{j=1}^{n}g\left(i,j\right)=0\; \end{array}$$

Seeing this contradiction, we arrive at the conclusion that (A7) has be true, which yields that

(A8) $$\begin{array}{c} f(x,x)=0\;\forall x\in R. \end{array}$$

Our next step is to prove that

(A9) $$\begin{array}{cc} f(x,y) & =f(y,x)\;\forall x,y\in R,\;\mathrm{and} \end{array}$$

(A10) $$\begin{array}{cc} f(x,y) & \neq 0\;\forall x,y\in R,x\neq y \end{array}$$

have to hold. Therefore, we inspect the symmetry condition of our metric using the same a,b as above, which tells us that

(A11) $$\begin{array}{c} D_{1}\left(a,b\right)=D_{1}\left(b,a\right)⟹D_{1}{\left(a,b\right)}^{p}=D_{1}{\left(b,a\right)}^{p}⟺\sum_{i=1}^{n}\sum_{j=1}^{n}f\left(a_{i},b_{j}\right)g\left(i,j\right)=\sum_{i=1}^{n}\sum_{j=1}^{n}f\left(b_{i},a_{j}\right)g\left(i,j\right)\\ \;⟺\sum_{i=1}^{n}\sum_{j=1}^{n}f\left(x,y\right)g\left(i,j\right)=\sum_{i=1}^{n}\sum_{j=1}^{n}f\left(y,x\right)g\left(i,j\right)⟺f\left(x,y\right)\sum_{i=1}^{n}\sum_{j=1}^{n}g\left(i,j\right)=f\left(y,x\right)\sum_{i=1}^{n}\sum_{j=1}^{n}g\left(i,j\right). \end{array}$$

Using the fact that (A7) holds, we can simplify to

(A12) $$\begin{array}{c} ⟺f(x,y)=f(y,x). \end{array}$$

Next, we aim for (A10): Since a≠b, we know because of the metric properties that

$$\begin{array}{c} D_{1}\left(a,b\right)>0⟹D_{1}{\left(a,b\right)}^{p}>0⟺\sum_{i=1}^{n}\sum_{j=1}^{n}f\left(a_{i},b_{j}\right)g\left(i,j\right)>0\\ \;⟺\sum_{i=1}^{n}\sum_{j=1}^{n}f\left(x,y\right)g\left(i,j\right)>0⟺f\left(x,y\right)\sum_{i=1}^{n}\sum_{j=1}^{n}g\left(i,j\right)>0. \end{array}$$

Using (A7), we can derive

⟺f(x,y)≠0∀x,y∈R,x≠y

We can use these proven properties of *f* to finally show that *g* has to have the aforementioned properties. Therefore, let $1\leq \hat{n},\hat{m}\leq n,\hat{m}\neq \hat{n}$. We define four vectors a,b,c,d∈R:

(A13) $$\begin{array}{cc} a & ={\left(x,x,\ldots ,x\underset{\mathrm{Position}\hat{n}}{\underset{︸}{,y,}}x,\ldots ,x\right)}^{T},\;b={\left(x,x,\ldots ,x\underset{\mathrm{Position}\hat{m}}{\underset{︸}{,y,}}x,\ldots ,x\right)}^{T},\\ c & ={\left(x,\ldots ,x\right)}^{T},\;d={\left(x,x,\ldots ,x\underset{\mathrm{Position}\hat{n}}{\underset{︸}{,y,}}x\ldots ,x\underset{\mathrm{Position}\hat{m}}{\underset{︸}{,y,}}x,\ldots ,x\right)}^{T}. \end{array}$$

Using the metric properties, we know that

$$\begin{array}{c} D_{1}\left(a,a\right)=0⟺D_{1}{\left(a,a\right)}^{p}=0⟺\sum_{i=1}^{n}\sum_{j=1}^{n}f\left(a_{i},a_{j}\right)g\left(i,j\right)=0\\ \;⟺\sum_{i=1,i\neq \hat{n}}^{n}\sum_{j=1,j\neq \hat{n}}^{n}\overset{=0\;(A8)}{\overset{︷}{f(x,x)}}g\left(i,j\right)+\sum_{i=1,i\neq \hat{n}}^{n}f\left(x,y\right)g\left(i,\hat{n}\right)+\sum_{j=1,j\neq \hat{n}}^{n}f\left(y,x\right)g\left(\hat{n},j\right)+\overset{=0\;(A8)}{\overset{︷}{f(y,y)}}g\left(\hat{n},\hat{n}\right)=0\\ \;⟺\sum_{i=1,i\neq \hat{n}}^{n}f\left(x,y\right)g\left(i,\hat{n}\right)+\sum_{j=1,j\neq \hat{n}}^{n}f\left(y,x\right)g\left(\hat{n},j\right)=0⟺\sum_{j=1,j\neq \hat{n}}^{n}f\left(y,x\right)g\left(\hat{n},j\right)=-\sum_{i=1,i\neq \hat{n}}^{n}f\left(x,y\right)g\left(i,\hat{n}\right)\\ \;⟺f\left(y,x\right)\sum_{j=1,j\neq \hat{n}}^{n}g\left(\hat{n},j\right)=-f\left(x,y\right)\sum_{i=1,i\neq \hat{n}}^{n}g\left(i,\hat{n}\right). \end{array}$$

Using the symmetry property (A9), the fact (A10) and renaming the indices, we get:

(A14) $$\begin{array}{c} ⟺f\left(x,y\right)\sum_{j=1,j\neq \hat{n}}^{n}g\left(\hat{n},j\right)=-f\left(x,y\right)\sum_{i=1,i\neq \hat{n}}^{n}g\left(i,\hat{n}\right)⟺\forall \hat{n}=1,\ldots ,n:\;\sum_{i=1,i\neq \hat{n}}^{n}g\left(\hat{n},i\right)=-\sum_{i=1,i\neq \hat{n}}^{n}g\left(i,\hat{n}\right) \end{array}$$

Since we know that $D_{1}$ is a metric, we see:

$$\begin{array}{cccc} \; & \; & D_{1}\left(a,c\right) & =D_{1}\left(c,a\right)⟹D_{1}{\left(a,c\right)}^{p}=D_{1}{\left(c,a\right)}^{p}\\ \; & ⟺ & \sum_{i=1,i\neq \hat{n}}^{n}\sum_{j=1}^{n}\overset{=0\;(A8)}{\overset{︷}{f(x,x)}}g\left(i,j\right)+\sum_{j=1}^{n}f\left(y,x\right)g\left(\hat{n},j\right) & =\sum_{i=1}^{n}\sum_{j=1,j\neq \hat{n}}^{n}\overset{=0\;(A8)}{\overset{︷}{f(x,x)}}g\left(i,j\right)+\sum_{i=1}^{n}f\left(y,x\right)g\left(i,\hat{n}\right)\\ \; & ⟺ & f\left(y,x\right)\sum_{j=1}^{n}g\left(\hat{n},j\right) & =f\left(x,y\right)\sum_{i=1}^{n}g\left(i,\hat{n}\right) \end{array}$$

Using the symmetry property (A9):

$$\begin{array}{ccc} ⟺ & \;\;\;\;\;\;\;\;\;\;f\left(y,x\right)\sum_{j=1}^{n}g\left(\hat{n},j\right) & =f\left(y,x\right)\sum_{i=1}^{n}g\left(i,\hat{n}\right) \end{array}$$

Using the fact (A10) and renaming the indices, we get

(A15) $$\begin{array}{c} ⟺\forall \hat{n}=1,\ldots ,n:\;\sum_{i=1}^{n}g\left(\hat{n},i\right)=\sum_{i=1}^{n}g\left(i,\hat{n}\right) \end{array}$$

Next, we add up/subtract the results (A14) and (A15):

$$\begin{array}{ccc} \; & (A14)+(A15): & 2\sum_{i=1,i\neq \hat{n}}^{n}g\left(\hat{n},i\right)+g\left(\hat{n},\hat{n}\right)=g\left(\hat{n},\hat{n}\right)\\ \; & (A14)-(A15): & g\left(\hat{n},\hat{n}\right)=2\sum_{i=1,i\neq \hat{n}}^{n}g\left(i,\hat{n}\right)+g\left(\hat{n},\hat{n}\right) \end{array}$$

subtracting $g(\hat{n},\hat{n})$ and dividing by 2 on both sides in both equations give us

(A16) $$\begin{array}{cc} \sum_{\begin{array}{c} i=1\\ i\neq \hat{n} \end{array}}^{n}g\left(\hat{n},i\right) & =0\;\forall \hat{n}=1,\ldots ,n. \end{array}$$

(A17) $$\begin{array}{cc} \sum_{\begin{array}{c} i=1\\ i\neq \hat{n} \end{array}}^{n}g\left(i,\hat{n}\right) & =0\;\forall \hat{n}=1,\ldots ,n. \end{array}$$

For the case n=2, we are done with the proof of (A4), as in this case both (A16) and (A17) read as

g(1,2)=0andg(2,1)=0.

Assume now that n≥3:

$$\begin{array}{c} D_{1}\left(a,b\right)=D_{1}\left(b,a\right)⟹D_{1}{\left(a,b\right)}^{p}=D_{1}{\left(b,a\right)}^{p}\\ \;⟺\sum_{\begin{array}{c} i=1\\ i\neq \hat{n} \end{array}}^{n}\sum_{\begin{array}{c} j=1\\ j\neq \hat{m} \end{array}}^{n}\overset{=0\;(A8)}{\overset{︷}{f(x,x)}}g\left(i,j\right)+\sum_{\begin{array}{c} j=1\\ j\neq \hat{m} \end{array}}^{n}f\left(y,x\right)g\left(\hat{n},j\right)+\sum_{\begin{array}{c} i=1\\ i\neq \hat{n} \end{array}}^{n}f\left(x,y\right)g\left(i,\hat{m}\right)+\overset{=0\;(A8)}{\overset{︷}{f(y,y)}}g\left(\hat{n},\hat{m}\right)\\ \;=\sum_{\begin{array}{c} i=1\\ i\neq \hat{m} \end{array}}^{n}\sum_{\begin{array}{c} j=1\\ j\neq \hat{n} \end{array}}^{n}\overset{=0\;(A8)}{\overset{︷}{f(x,x)}}g\left(i,j\right)+\sum_{\begin{array}{c} j=1\\ j\neq \hat{n} \end{array}}^{n}f\left(y,x\right)g\left(\hat{m},j\right)+\sum_{\begin{array}{c} i=1\\ i\neq \hat{m} \end{array}}^{n}f\left(x,y\right)g\left(i,\hat{n}\right)+\overset{=0\;(A8)}{\overset{︷}{f(y,y)}}g\left(\hat{m},\hat{n}\right)\\ \;⟺\sum_{\begin{array}{c} j=1\\ j\neq \hat{m} \end{array}}^{n}f\left(y,x\right)g\left(\hat{n},j\right)+\sum_{\begin{array}{c} i=1\\ i\neq \hat{n} \end{array}}^{n}f\left(x,y\right)g\left(i,\hat{m}\right)=\sum_{\begin{array}{c} j=1\\ j\neq \hat{n} \end{array}}^{n}f\left(y,x\right)g\left(\hat{m},j\right)+\sum_{\begin{array}{c} i=1\\ i\neq \hat{m} \end{array}}^{n}f\left(x,y\right)g\left(i,\hat{n}\right) \end{array}$$

Using the symmetry property (A9) and the fact (A10), we can pull out a factor f(x,y) in all sums and divide by it. Moreover, we rename the indices and get:

(A18) $$\begin{array}{c} ⟺\sum_{\begin{array}{c} i=1\\ i\neq \hat{m} \end{array}}^{n}g\left(\hat{n},i\right)+\sum_{\begin{array}{c} i=1\\ i\neq \hat{n} \end{array}}^{n}g\left(i,\hat{m}\right)=\sum_{\begin{array}{c} i=1\\ i\neq \hat{n} \end{array}}^{n}g\left(\hat{m},i\right)+\sum_{\begin{array}{c} i=1\\ i\neq \hat{m} \end{array}}^{n}g\left(i,\hat{n}\right). \end{array}$$

Use the facts (A16) and (A17) that

(A19) $$\begin{array}{c} \sum_{\begin{array}{c} i=1\\ i\neq \hat{n} \end{array}}^{n}g\left(\hat{n},i\right)=0,\;\sum_{\begin{array}{c} i=1\\ i\neq \hat{m} \end{array}}^{n}g\left(\hat{m},i\right)=0,\;\sum_{\begin{array}{c} i=1\\ i\neq \hat{n} \end{array}}^{n}g\left(i,\hat{n}\right)=0,\;\sum_{\begin{array}{c} i=1\\ i\neq \hat{m} \end{array}}^{n}g\left(i,\hat{m}\right)=0. \end{array}$$

We subtract the first and fourth expression from (A19) from the left hand side of (A18), and the second and third expression from (A19) from the right-hand side of (A18), which gives us

$$g\left(\hat{n},\hat{n}\right)-g\left(\hat{n},\hat{m}\right)+g\left(\hat{m},\hat{m}\right)-g\left(\hat{n},\hat{m}\right)=g\left(\hat{m},\hat{m}\right)-g\left(\hat{m},\hat{n}\right)+g\left(\hat{n},\hat{n}\right)-g\left(\hat{m},\hat{n}\right),$$

which simplifies to

(A20) $$\begin{array}{cc} g(\hat{n},\hat{m}) & =g\left(\hat{m},\hat{n}\right)\;\forall \hat{m}\neq \hat{n},\;1\leq \hat{m},\hat{n}\leq n. \end{array}$$

Finally, we look at $D_{1}\left(d,d\right)$:

$$\begin{array}{cc} \; & D_{1}\left(d,d\right)=0⟺D_{1}{\left(d,d\right)}^{p}=0\\ ⟺ & \sum_{\begin{array}{c} i=1\\ i\neq \hat{n},\hat{m} \end{array}}^{n}\sum_{\begin{array}{c} j=1\\ j\neq \hat{n},\hat{m} \end{array}}^{n}\overset{=0\;(A8)}{\overset{︷}{f(x,x)}}g\left(i,j\right)+\sum_{\begin{array}{c} i=1\\ i\neq \hat{n},\hat{m} \end{array}}^{n}f\left(x,y\right)g\left(i,\hat{n}\right)+\sum_{\begin{array}{c} i=1\\ i\neq \hat{n},\hat{m} \end{array}}^{n}f\left(x,y\right)g\left(i,\hat{m}\right)\\ \; & +\sum_{\begin{array}{c} j=1\\ j\neq \hat{n},\hat{m} \end{array}}^{n}f\left(y,x\right)g\left(\hat{n},j\right)+\sum_{\begin{array}{c} j=1\\ j\neq \hat{n},\hat{m} \end{array}}^{n}f\left(x,y\right)g\left(\hat{m},j\right)+\overset{=0\;(A8)}{\overset{︷}{f(y,y)}}\left(g\left(\hat{n},\hat{n}\right)+g\left(\hat{n},\hat{m}\right)+g\left(\hat{m},\hat{n}\right)+g\left(\hat{m},\hat{m}\right)\right)=0\\ ⟺ & \sum_{\begin{array}{c} i=1\\ i\neq \hat{n},\hat{m} \end{array}}^{n}f\left(x,y\right)g\left(i,\hat{n}\right)+\sum_{\begin{array}{c} i=1\\ i\neq \hat{n},\hat{m} \end{array}}^{n}f\left(x,y\right)g\left(i,\hat{m}\right)+\sum_{\begin{array}{c} j=1\\ j\neq \hat{n},\hat{m} \end{array}}^{n}f\left(y,x\right)g\left(\hat{n},j\right)+\sum_{\begin{array}{c} j=1\\ j\neq \hat{n},\hat{m} \end{array}}^{n}f\left(x,y\right)g\left(\hat{m},j\right)=0. \end{array}$$

As above, we use the symmetry property (A9) and the fact (A10), pull out a factor f(x,y) in all sums and divide by it. Moreover, we rename the indices and get:

(A21) $$\begin{array}{cc} \sum_{\begin{array}{c} i=1\\ i\neq \hat{n},\hat{m} \end{array}}^{n}g\left(i,\hat{n}\right)+\sum_{\begin{array}{c} i=1\\ i\neq \hat{n},\hat{m} \end{array}}^{n}g\left(i,\hat{m}\right)+\sum_{\begin{array}{c} i=1\\ i\neq \hat{n},\hat{m} \end{array}}^{n}g\left(\hat{n},i\right)+\sum_{\begin{array}{c} i=1\\ i\neq \hat{n},\hat{m} \end{array}}^{n}g\left(\hat{m},i\right) & =0. \end{array}$$

Subtracting all four expressions in (A19) from (A21), we get:

(A22) $$\begin{array}{cc} -g\left(\hat{m},\hat{n}\right)-g\left(\hat{n},\hat{m}\right)-g\left(\hat{n},\hat{m}\right)-g\left(\hat{m},\hat{n}\right) & =0\\ ⟺g(\hat{n},\hat{m}) & =-g\left(\hat{m},\hat{n}\right)\;\forall \hat{m}\neq \hat{n},\;1\leq \hat{m},\hat{n}\leq n. \end{array}$$

Adding up (A20) + (A22) gives us

(A23) $$\begin{array}{cc} g(\hat{n},\hat{m}) & =0\;\forall \hat{m}\neq \hat{n},\;1\leq \hat{m},\hat{n}\leq n. \end{array}$$

which concludes our proof of (A4) for n≥3. To finally show (A2), we have to prove (A5) for n>1. As we already showed that g(i,j) has to be 0 for i≠j, we assume that $D_{1}$ takes the following form:

(A24) $$\begin{array}{c} D_{1}{\left(a,b\right)}^{p}=\sum_{i=1}^{n}f\left(a_{i},b_{i}\right)g\left(i,i\right). \end{array}$$

We define $a={\left(x,x,\ldots ,x\right)}^{T}$ as above and two more vectors *b* and *c*:

$$b={\left(x,x,\ldots ,x\begin{array}{c} \underset{︸}{,y,}\\ \mathrm{Position}\;i \end{array}x,\ldots ,x\right)}^{T},c={\left(x,x,\ldots ,x\begin{array}{c} \underset{︸}{,y,}\\ \mathrm{Position}\;j \end{array}x,\ldots ,x\right)}^{T}$$

We now prove (A5) by showing that the opposite assumptions lead to a contradiction. Assume therefore that (A5)’ holds:

$$\begin{array}{c} \exists i,j,i\neq j:g(i,i)\leq 0\wedge g(j,j)\geq 0 \end{array}$$

As we assume $D_{1}$ to be a metric, we know that $D{\left(a,b\right)}^{p}>0\wedge D{\left(a,c\right)}^{p}>0$. Using (A7) and the simplified form of $D_{1}$ (A24), we see that the following statement has to hold:

(A25) $$\begin{array}{cc} f(x,y)g(i,i)>0 & \wedge \;f(x,y)g(j,j)>0 \end{array}$$

Inspecting (A25), we see that neither g(i,i)=0 nor g(j,j)=0 can fulfil these inequalities, thus we assume g(i,i)<0∧g(j,j)>0. Using these assumptions and dividing by g(i,i) resp. g(j,j), we arrive at:

(A26) $$\begin{array}{c} f(x,y)<0\wedge f(x,y)>0 \end{array}$$

As we arrived at a contradictory result (A26) by assuming (A5)’ to hold, we can conclude that (A5) has to hold and therefore we have proven (A2). □
